# Cross-Scale Analysis of the Region Effect on Vascular Plant Species Diversity in Southern and Northern European Mountain Ranges

**DOI:** 10.1371/journal.pone.0015734

**Published:** 2010-12-22

**Authors:** Jonathan Lenoir, Jean-Claude Gégout, Antoine Guisan, Pascal Vittoz, Thomas Wohlgemuth, Niklaus E. Zimmermann, Stefan Dullinger, Harald Pauli, Wolfgang Willner, John-Arvid Grytnes, Risto Virtanen, Jens-Christian Svenning

**Affiliations:** 1 Ecoinformatics and Biodiversity Group, Department of Biological Sciences, Aarhus University, Aarhus, Denmark; 2 AgroParisTech, Unité Mixte de Recherche 1092 AgroParisTech-Institut National de la Recherche Agronomique (INRA), Laboratoire d'Etude des Ressources Forêt-Bois (LERFoB), Nancy, France; 3 Center for Advanced Studies in Ecology and Biodiversity (CASEB), Departamento de Ecologia, Pontificia Universidad Católica de Chile, Santiago, Chile; 4 Faculty of Biology and Medicine, Department of Ecology and Evolution, University of Lausanne, Lausanne, Switzerland; 5 Faculty of Geosciences and Environment, University of Lausanne, Lausanne, Switzerland; 6 Swiss Federal Research Institute for Forest, Snow and Landscape Research (WSL), Birmensdorf, Switzerland; 7 Vienna Institute for Nature Conservation and Analyses, Vienna, Austria; 8 Faculty Centre for Biodiversity, Department of Conservation Biology, Vegetation and Landscape Ecology, University of Vienna, Vienna, Austria; 9 Institute of Mountain Research: Man and the Environment (IGF) of the Austrian Academy of Sciences, Faculty Centre for Biodiversity, Department of Conservation Biology, Vegetation and Landscape Ecology, University of Vienna, Vienna, Austria; 10 Department of Biology, University of Bergen, Bergen, Norway; 11 Department of Biology, University of Oulu, Oulu, Finland; Umea University, Sweden

## Abstract

**Background:**

The divergent glacial histories of southern and northern Europe affect present-day species diversity at coarse-grained scales in these two regions, but do these effects also penetrate to the more fine-grained scales of local communities?

**Methodology/Principal Findings:**

We carried out a cross-scale analysis to address this question for vascular plants in two mountain regions, the Alps in southern Europe and the Scandes in northern Europe, using environmentally paired vegetation plots in the two regions (n = 403 in each region) to quantify four diversity components: (i) total number of species occurring in a region (total γ-diversity), (ii) number of species that could occur in a target plot after environmental filtering (habitat-specific γ-diversity), (iii) pair-wise species compositional turnover between plots (plot-to-plot β-diversity) and (iv) number of species present per plot (plot α-diversity). We found strong region effects on total γ-diversity, habitat-specific γ-diversity and plot-to-plot β-diversity, with a greater diversity in the Alps even towards distances smaller than 50 m between plots. In contrast, there was a slightly greater plot α-diversity in the Scandes, but with a tendency towards contrasting region effects on high and low soil-acidity plots.

**Conclusions/Significance:**

We conclude that there are strong regional differences between coarse-grained (landscape- to regional-scale) diversity components of the flora in the Alps and the Scandes mountain ranges, but that these differences do not necessarily penetrate to the finest-grained (plot-scale) diversity component, at least not on acidic soils. Our findings are consistent with the contrasting regional Quaternary histories, but we also consider alternative explanatory models. Notably, ecological sorting and habitat connectivity may play a role in the unexpected limited or reversed region effect on plot α-diversity, and may also affect the larger-scale diversity components. For instance, plot connectivity and/or selection for high dispersal ability may increase plot α-diversity and compensate for low total γ-diversity.

## Introduction

The mechanisms that shape species diversity fascinated naturalists 150–200 years ago and continue to form one of the main questions in 21^st^-century science [1]. Many studies have singled out environmental factors such as current climate and topographic heterogeneity as the primary determinants of species richness [2]–[5]. However, historical factors such as past climate and postglacial re-colonisation have also been widely reported as important species richness determinants [2], [6]–[8]. Numerous comparisons of species richness in environmentally similar regions with different long-term biogeographic histories have revealed substantial differences in species richness [9]–[13]. Most comparative studies have used range-map-based and atlas-based data to estimate and compare the number of species co-occurring in a given pair of environmentally similar grid cells, usually 10^1^ to >10^6^ km^2^ in size [12]. Given the generally coarse resolution of these studies, a question that arises is whether substantial differences in species richness between environmentally similar sampling units from regions of differing history also penetrate to the more fine-grained scales of local communities. The dearth of fine-grained studies impedes disentangling historical effects from the effects of potentially varying levels of environmental heterogeneity on species diversity [13]. To understand how biogeographic history affects species diversity, Ricklefs *et al.* [13] proposed a complete deconstruction of the mesoscale species richness (γ-diversity) component into its compositional species turnover (β-diversity) and local species richness (α-diversity) components in regions with different historical backgrounds. This approach would require comparisons among environmentally similar sites at a range of spatial resolutions. The region effect on γ-, β- and α-diversity have hitherto rarely been simultaneously analysed in comparative studies [14].

It is generally accepted that γ-diversity is determined not only by the current environment but also by long-term historical factors, i.e., ultimately immigration, speciation and extinction [10]–[13], [15]–[18]. For example, geographic variation in the magnitude of the recurrent dramatic climatic shifts during the Pleistocene has been proposed to have exerted a strong influence on extinction and speciation and thus also on current species diversity patterns [19], [20]. In line with this suggestion, Svenning *et al.* [7] found that plant species richness at a grain size of 2500 km^2^ was more strongly related to topographic heterogeneity in southern Europe, where the influence of the Pleistocene glacial maxima had been weaker than in northern Europe. However, given the study's coarse grain, geographic variability in the current environment could not be excluded as a driver of this pattern. In particular, the habitats added with increasing topographic heterogeneity could simply be more species rich in southern than in northern Europe [7].

Substantial differences in β- and α-diversity between regions may also result from different historical filters [2], [4], [21], [22]. Concerning β-diversity, Graham *et al.* [21] showed that historical patterns of habitat connectivity best explain contemporary patterns of rainforest fauna turnover across northeast Australia. Concerning α-diversity, Leathwick *et al.* [2] concluded in an analysis of tree plots that, in addition to productivity, history is also an important determinant of tree α-diversity in New Zealand. However, none of these studies compared similar environmental sites from regions of differing history to assess directly the region effect on β- and α-diversity.

Here, we investigated the region effect on vascular plant diversity across spatial scales by comparing γ-, β- and α-diversity in the Alps (southern Europe) and the Scandes (northern Europe) mountain ranges using fine-grained (<1000 m^2^) vegetation plot data from environmentally paired sites in the two regions. These two regions have experienced markedly different Quaternary histories. Notably, the Alps were never completely glaciated during the Pleistocene (reflecting their southern location and highly dissected topography) and were close to the main glacial refugia of the European flora in southern Europe and the peripheral alpine refugia around the margin of the Alps [23]–[26]. By contrast, the Scandes were almost completely glaciated during the Pleistocene glacial maxima (reflecting their northern location and less dissected topography) and located distant from the southern-European glacial refugia [23], [24], [27] (although some species survived in more northern refugia [28]). We note that the two regions also are different in terms of macroclimate, geology and Holocene land-use histories, with the Alps encompassing a larger climatic variation, having a more heterogeneous geological structure and experiencing a more intensive land use for a longer time than the Scandes. We compared the vascular plant diversity of the two mountain ranges, defining four diversity components for the purpose of this study: (i) the total set of species occurring in a given region (total γ-diversity); (ii) the subset of this regional flora that can tolerate the environmental conditions in a specific habitat (habitat-specific γ-diversity) [17], [18]; (iii) the pair-wise compositional dissimilarity [29], [30] between vegetation plots (plot-to-plot β-diversity, cf. [29], [30] for a thorough discussion of β-diversity measures and terminology); and (iv) species richness in single vegetation plots (plot α-diversity). Additionally, we compared the vascular plant flora of the Alps and the Scandes with respect to the relationship between two of these diversity components, plot α-diversity and habitat-specific γ-diversity, known as the local-to-regional species richness relationship [17], [18], [31]. Using these measures in a cross-scale framework and accounting for the effects of environmental differences in these diversity components between the two studied regions, we tested the following three hypotheses concerning the region effect on vascular plant diversity:

- Total and habitat-specific γ-diversity should be higher in the Alps than in the Scandes because of higher rates of postglacial re-colonisation as well as greater possibilities for *in-situ* glacial survival and speciation in the Alps due to a greater proximity to glacial refugia and more widespread and diverse ice-free nunatak areas.
- Plot-to-plot β-diversity from within small localities to across regions should also be higher in the Alps than in the Scandes because of a geologically and geographically more heterogeneous set of refugial re-colonisation sources as well as a greater habitat fragmentation resulting from patchy geologic features and Holocene land-use histories in the Alps.
- Assuming that the above-mentioned assumptions of the effects of divergent glacial histories on species diversity also penetrate to the more fine-grained scales of local communities, plot α-diversity should be higher in the Alps than in the Scandes.

## Methods

### Methods overview

We first gathered as much vegetation-plot data as possible in the Alps and the Scandes to capture a sufficient amount of habitat diversity and to allow reasonable overlap of environmental conditions between both regions. We then ran a selection procedure to pair plots in the Scandes with environmentally similar plots in the Alps. Based on these environmentally paired plots, we subsequently explored and tested our three hypotheses separately using several analytical techniques. Finally, we analysed and compared the relationship of plot α-diversity to habitat-specific γ-diversity in the Alps and the Scandes.

### Floristic data

A total of 32,013 vegetation plots in habitats from lowland forests to alpine grasslands were gathered from published and unpublished sources for the Alps: France, *n* = 12,666 [32], [33], Switzerland, *n* = 13,818 [34]–[36] and Austria, *n* = 4326 [37], [38]; and the Scandes: Norway, *n* = 996 [39], [40] and Finland, *n* = 207 [41]. All plots were imported to TURBOVEG [42]. During the import procedure, all vascular taxa were linked to TURBOVEG's European species list, a list of valid names and synonyms based on Flora Europaea [43]. We updated this list by adding taxa and synonyms not yet included. By relating all vegetation plots to this updated list, we ensured that the nomenclature was consistent. Structural vegetation layers of each plot were combined to avoid counting taxa more than once. For the purpose of computing the different diversity components, we pooled sub-specific taxa and excluded records identified only to the genus level, thereby focusing solely on the species level.

### Environmental data

Ellenberg's indicator system [44] can be used to estimate environmental conditions for vegetation plots [45]–[47] and has successfully been applied in many ecological studies across Europe [48]–[52]. Ellenberg *et al.* [44] ranked most of the plant taxa of Central Europe according to their occurrence optimum along key environmental gradients for plants (L, light; T, temperature; K, continentality; F, soil moisture; R, soil pH; N, soil fertility; henceforth termed Ellenberg's indicator factors) using an ordinal scale ranging from 1 (lowest) to 9 (highest) in terrestrial environments. No values were assigned to plant taxa estimated to be indifferent for a given environmental gradient. To estimate the value of an environmental variable for a given plot, the indicator values of all taxa present in the plot are commonly averaged (excluding taxa that lack indicator values) [44] and we followed this approach here. For the Alps (*n* = 30,810 plots), we used the original indicator values because of the close geographical proximity of the Alps to the region that was the focus of Ellenberg's indicator system. For the Scandes (*n* = 1203 plots), the indicator values were adjusted following Diekmann [45] to correct for regional deviations [46]. We used all plots available in the Scandes and focused on taxa that had a frequency of ≥5% in the data set to run the procedure detailed by Diekmann [45], thus replacing the original indicator value with the re-calculated optimum value. Table S1 lists taxa that either had no original values (coded as indifferent by Ellenberg *et al.* [44]) and to which we assigned indicator values or for which adjusted values differed from original values. All indicator value computations were carried out in TURBOVEG.

### Plot selection procedure

We then selected all plots that matched the following three criteria: (i) The plots were referenced in time (year) and space (longitude, latitude, and altitude), were sampled during the period 1909–2009 and had unique geographical coordinates (when several plots had the same geographical coordinates, one was randomly selected); (ii) for each Ellenberg's indicator factor in each plot, at least five taxa with Ellenberg's indicator values were present to allow for a reliable estimation [46]; and (iii) the plot size was known and was within a range of 0.1–1000 m^2^, restricting the study to fine-grained vegetation plots. A total of 11,249 plots in the Alps (5°–16°30′ E, 43°45′–48°30′ N, 50–4000 m altitude) and 481 plots in the Scandes (5°–27°30′ E, 58°30′–71° N, 50–2000 m altitude) met these criteria.

Before analysing the diversity components, we selected a subset of environmentally similar paired plots from the Alps and the Scandes using a principal component analysis (PCA) on all six of Ellenberg's indicator factors as well as plot size for all 11,249 plots in the Alps and then added the 481 plots from the Scandes (see Text S1 for a detailed description of the pairing procedure). This procedure allowed us to arrange the 481 plots from the Scandes in the environmental space defined by light, temperature, continentality, soil moisture, soil pH, soil fertility and plot size conditions observed in the 11,249 plots from the Alps (Figure S1). Hereby, plots sharing similar environmental conditions were arranged close to each other in the environmental space of the PCA, and thus the closest Alps-Scandes pairs within this environmental space were considered environmentally similar. In this procedure, it was important to use as many vegetation plots as possible to increase the potential for finding plot pairs with closely similar environmental conditions. The PCA was performed in R [53] using the ade4 package. A total of 403 Alps–Scandes pairs were found to be sufficiently similar (Text S1) and thus selected for use in the diversity analyses (Figure S1C). Most of the selected plots had been sampled during the last decades in both the Alps (first quartile: 1972, median: 1994, third quartile: 1994) and the Scandes (first quartile: 1997, median: 2000, third quartile: 2003). The plots were geographically well distributed across the Alps, extending from southern France to north-eastern Austria. The plots in the Scandes were more clustered but were nevertheless located in different mountain areas from southern Norway to north-western Finland. Displaying the selected plots along the indicator values separately showed that the 403 Alps–Scandes pairs were sampled in similar conditions for all six Ellenberg's indicator factors (Figure S2). Generally, Ellenberg's indicator factors indicated that the plots were sampled in cold climates and acidic soils with slightly more open and acidic habitats in the Alps than in the Scandes and slightly more continental and humid habitat in the Scandes than in the Alps (Figure S1). Plot size was slightly larger in the Alps (first quartile: 4 m^2^, median: 30 m^2^, third quartile: 100 m^2^) than in the Scandes (first quartile: 4 m^2^, median: 25 m^2^, third quartile: 25 m^2^). To account for these imperfections in the pairing, we used the six Ellenberg's indicator factors and plot size or transformations of these variables as covariates in the subsequent models of β- and α-diversity. The main vegetation types selected in the 403 Alps–Scandes pairs were subalpine-open-woodland, alpine-grassland and alpine-heath-like communities within which the most frequent species are respectively: *Homogyne alpina*, *Leontodon pyrenaicus* and *Vaccinium myrtillus* in the Alps and *Deschampsia flexuosa*, *Polygonum viviparum* and *Empetrum nigrum* in the Scandes.

### Statistical analyses

#### Total and habitat-specific γ-diversity

Having defined pairs of environmentally similar plots, we first compared total and habitat-specific γ-diversity in the Alps and the Scandes to evaluate our first hypothesis that total and habitat-specific γ-diversity are higher in the Alps than in the Scandes. Total γ-diversity values in the Alps and the Scandes were determined from the lists of all species occurring in the 403 Alps–Scandes pairs of plots.

To estimate the size of the habitat-specific γ-diversity, we intersected the total γ-diversity in a region with the lists of all species bound to a certain set of environmental conditions, following the approach proposed by Ewald [54], who used Beals' index. Beals' index estimates the probability of encountering a given species in a given plot from the actual species composition in the plot and the pattern of species co-occurrence in the whole floristic matrix of *r* rows (plots) and *p* columns (species):
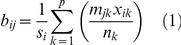
where *b_ij_* is the estimated probability of species *j* to occur in plot *i*, *s_i_* the number of species in plot *i*, *m_jk_* the number of joint occurrences of species *j* and *k*, *n_k_* the number of occurrences of species *k* in the matrix and *x_ik_* a binary value either equal to one or zero depending on the corresponding presence or absence of species *k* in plot *i*. The whole floristic matrix was built by merging all 806 plots from the two regions. For each plot and species in this matrix, we then calculated the probability *b_ij_*. Therefore, all plots were associated with a vector of *p b_ij_* values. Finally, to estimate the habitat-specific γ-diversity for each of the 806 plots we intersected the list of all species occurring in its region with the list of all species with a probability *b_ij_* exceeding a certain cut-off value for the plot [54]. Thus, only species occurring in a given region could contribute to the estimation of the size of the habitat-specific γ-diversity for each plot in that region. By increasing the cut-off probability *b_ij_*, we increased the strength of environmental filtering [54]. However, highly frequent species tend to have high *b_ij_* values because many species often co-occur with highly frequent species, whereas *b_ij_* is usually low for rare species [55]. This relationship could influence the size of the habitat-specific γ-diversity regardless of species' environmental preferences, especially for high values of the cut-off probability *b_ij_*, which are more likely to include only the most frequent species in the habitat-specific γ-diversity.

Because only environmental filtering and not the relative overall frequency of each species should influence the size of habitat-specific γ-diversity, we re-computed the probability *b_ij_* (0<*b_ij_*<1) relative to the overall frequency *f_j_* (0<*f_j_*<1) of species *j* in the whole matrix:
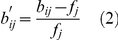



Note that *b_ij_*' does not represent a probability value anymore and can reach values >1 and <0. The fundamental logic behind equation 2 is to give more weight to rare species: for a given *b_ij_* value the lower the denominator (*f_j_*), the larger the increase in *b_ij_*', i.e., emphasising rare species over frequent ones. Since we computed *b_ij_*' across the whole matrix (including all species and plots from the two regions), there was no bias in the comparison of the two regions with regard to species frequency. We estimated the size of the habitat-specific γ-diversity for each plot in the Alps and the Scandes by choosing the cut-off value for *b_ij_*' that maximised the strength of environmental filtering (high values of *b_ij_*') without lowering the size of the habitat-specific γ-diversity below the observed species richness in any of the plots in the Alps and the Scandes. In other words, we iteratively increased the cut-off value for *b_ij_*' until the estimated size of the habitat-specific γ-diversity of at least one of the 806 plots fell below the observed species richness of that plot, and we chose the former iteration as a cut-off value for *b_ij_*'. We then tested whether the estimated size of the habitat-specific γ-diversity per plot was greater in the Alps than in the Scandes using a one-tailed paired-sample *t*-test.

#### Plot-to-plot β-diversity

We compared plot-to-plot β-diversity in the Alps and the Scandes to evaluate our second hypothesis that plot-to-plot β-diversity is higher in the Alps than in the Scandes. Plot-to-plot β-diversity was quantified across each region, thereby spanning a large range of spatial scales from within small localities to across regions. We used a pair-wise compositional dissimilarity index:
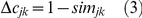


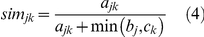
where *sim_jk_* is Simpson's similarity index computed between plot pairs (*j* ≠ *k*), *a_jk_* the number of species shared by both plots *j* and *k*, *b_j_* the number of species unique to plot *j* and *c_k_* the number of species unique to plot *k*. This approach yields a plot-to-plot measure of species turnover and corresponds to the pair-wise approach of compositional dissimilarities in the terminology of Tuomisto [29], [30]. To disentangle the true turnover in species composition from differences in species richness [56], we calculated Simpson's similarity index rather than the widely used Sørensen's similarity index. We are aware that using Simpson's similarity index might not measure true β-diversity as defined by Tuomisto [30], but our purpose here was not to quantify true β-diversity but rather to compare a specific aspect of β-diversity, i.e., species turnover, between the Alps and the Scandes. We first computed the average of all Δ*c_jk_* values in each region. For each sampling plot, we also computed the average of all Δ*c_jF_* values between the focal sampling plot *F* and all other 402 *j* plots in a given region (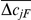) [30] to compare compositional distinctness in each Alps–Scandes pair. We then tested whether 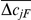 was higher in the Alps than in the Scandes using a one-tailed paired-sample *t*-test.

For each of the two regions, multiple linear regressions on distance matrices (MRM) [57], [58] were then used to analyse the dependency of plot-to-plot β-diversity on geographical distances between plots, after accounting for environmental distances. The plot values for each of the six Ellenberg's indicator factors as well as plot size were used to compute seven separate Euclidian distance matrices in each of the two regions. To reduce positive skewness, all seven distance matrices were log(x+1)-transformed before being used as explanatory distance matrices in the MRM analyses. For the geographical distance matrix, we used the latitudinal, longitudinal and altitudinal coordinates in the European Equidistant Conic projection to calculate Euclidian distances in metres between all possible plot pairs in a given region. Once the distance matrices had been unfolded into vectors of each unique pair, the MRM calculations were simply ordinary least square (OLS) multiple linear regressions, except that significance testing was performed by 10,000 permutations of the elements in the response matrix that maintain the dependence structure among the plots [57], [58]. The multiple regression analyses were done using backward elimination to retain only explanatory distance matrices with a statistically significant contribution (*P*<0.05). For each of the two regions, we accounted for environmental distances between plots by first fitting the best model of the floristic similarity matrix (*sim_jk_*) against the six Ellenberg's distance matrices and the plot size distance matrix. Then, we fitted the residuals of these models against geographical distance. To test the significance of the differences in the rate of distance decay in floristic similarity between different regions, Baselga [56] bootstrapped the coefficients of these regressions using the ordinary non-parametric bootstrap with a case re-sampling approach based on 1000 randomisations. Following this approach, we tested whether the intercept of these regressions of the residuals of the environmental models against geographical distance was greater in the Scandes (lower plot-to-plot β-diversity at short distance) than in the Alps and whether the slope was greater in the Alps (higher increase in plot-to-plot β-diversity with increasing distance) than in the Scandes. As the *P* values of the respective null hypotheses, we used the proportion of the 1000 iterations for which the intercept in the Alps was larger or the slope smaller, respectively, than the equivalent coefficients in the Scandes. We note that the results were quite similar if we instead fitted for each of the two regions the full model of *sim_jk_* against geographical distance as a main effect and all six Ellenberg's distance matrices and the plot size distance matrix as covariates in the model (results not shown).

#### Plot α-diversity

To evaluate our third hypothesis that plot α-diversity is higher in the Alps than in the Scandes, we compared plot α-diversity between the two regions, measured as the number of vascular plant species per plot, using a one-sided paired t-test. In addition, we carried out an OLS analysis of covariance (ANCOVA) to explore plot α-diversity in the two regions, with region as a main effect and the independent environmental variables (the six Ellenberg's indicator factors and plot size) as covariates. We used backward elimination to retain only significant (P<0.05) covariates in the ANCOVA. To reduce positive skewness in plot α-diversity after pooling the Alps and the Scandes data (n = 806 plots), we log-transformed plot α-diversity before using it as a dependent variable in the ANCOVA. We tested for spatial autocorrelation in the ANCOVA residuals using a Moran's I correlogram [59]. Significance was evaluated by 1000 permutations for each distance class with correction of the resulting P values for multiple comparisons using the Holm adjustment. Because there was significant spatial autocorrelation in the first distance classes, we used a spatial-error simultaneous autoregressive model, known as one of the most reliable error models [60].

We also tested for trends in the Alps–Scandes paired differences in plot α-diversity against environmental variables. To do so, we carried out an OLS model with “plot α-diversity in the Alps – plot α-diversity in the Scandes” as the dependent variable and the six Ellenberg's indicator factors and plot size for the Alps plots as independent variables (the Alps data set constituted the reference in the initial pairing procedure).

#### The relationship of plot α-diversity to habitat-specific γ-diversity

For each region, the significance of the relationship of plot α-diversity to habitat-specific γ-diversity was tested using a Monte Carlo test [17] to compare these two non-independent variables. In this procedure, the independent variable (X) was the habitat-specific γ-diversity in a given region for each plot in that region. For the dependent variable (Y), we drew a random value of the plot α-diversity from a uniform distribution so that 0≤Y≤X for each plot and calculated the correlation coefficient r between Y and X for all plots. This last step was repeated 10,000 times and the distribution of the 10,000 r values for the randomised data was compared to the empirical r between plot α-diversity and habitat-specific γ-diversity in the non-randomised data. The proportion of the 10,000 iterations where r from the randomised data exceeded r in the non-randomised data was used as an estimate of the P value of the null hypothesis.

We also tested the difference in the relationship of plot α-diversity to habitat-specific γ-diversity between the Alps and the Scandes by comparing the slope coefficients of plot α-diversity against habitat-specific γ-diversity in both areas. To test the significance of the difference in the slope coefficients between the Alps and the Scandes, we bootstrapped the coefficients of these two regressions using the ordinary non-parametric bootstrap with a case re-sampling approach based on 1000 randomisations. The proportion of bootstrapped slope estimates in the Alps overlapping with the distribution of bootstrapped slope estimates in the Scandes served as the *P* value estimate for a slope difference between the two regions.

All statistical analyses were carried out in R [53], using the boot, ecodist, ncf, spdep and vegan packages.

## Results

### Total and habitat-specific γ-diversity

In the 403 pairs of plots from the Alps and the Scandes, we found 675 species, 161 of which were present in both regions. Altogether for the environmentally paired plots, the total γ-diversity in the Alps (*n* = 565 species) was much greater than in the Scandes (*n* = 271 species), with 404 species unique to the Alps and 110 unique to the Scandes.

**Figure 1:**
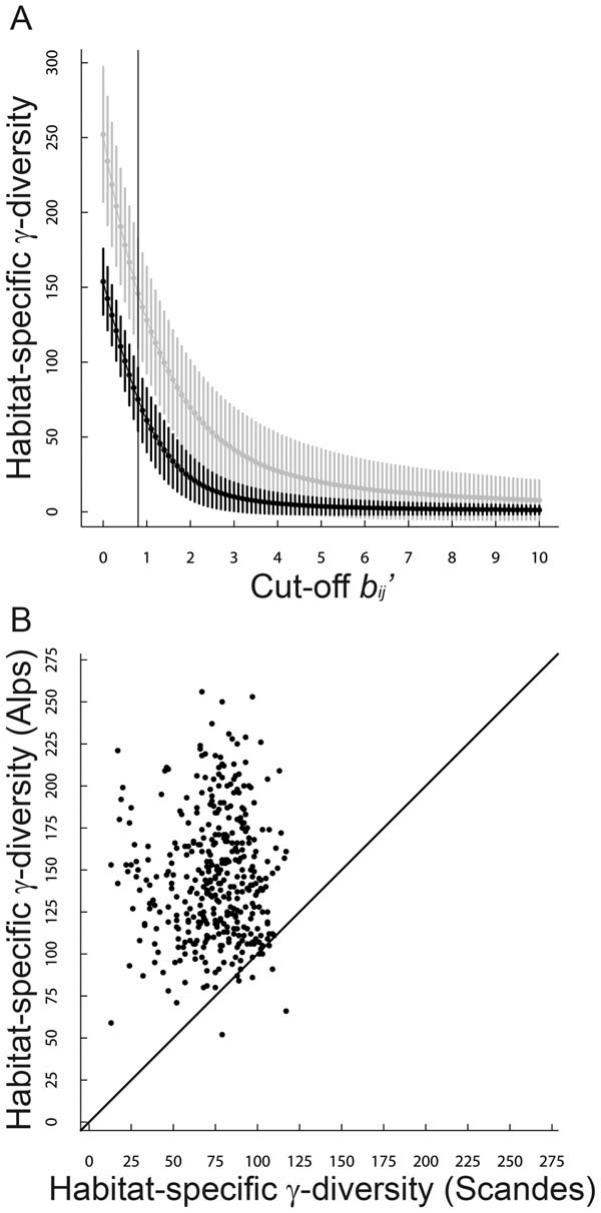
The region effect on habitat-specific γ-diversity. (**A**) Estimation of the size of the habitat-specific γ-diversity in the Alps and the Scandes with varying cut-off values based on the use of the modified Beals' index (*b_ij_*'). (**B**) Size of the habitat-specific γ-diversity in the Alps and the Scandes for a specific cut-off value *b_ij_*' = 0.80 that maximises the strength of the environmental filter without lowering the estimated size of the habitat-specific γ-diversity below the observed species richness in any of the plots. Curves show mean values of the size of the habitat-specific γ-diversity in the Alps (gray curve) and in the Scandes (dark curve), vertical bars show standard deviations in the Alps (gray vertical bars) and in the Scandes (dark vertical bars) and vertical lines indicate the maximum cut-off values of *b_ij_*' for meaningful estimation of the size of the habitat-specific γ-diversity.

The size of the habitat-specific γ-diversity, estimated by the modified Beals' index (*b_ij_*'), declined precipitously with an increasing cut-off value (Figure 1A). At *b_ij_*' cut-offs >0.80, at least one plot in the Scandes had the estimated size of its habitat-specific γ-diversity, dropping below its own plot α-diversity and larger cut-off levels were therefore not considered meaningful. Within the meaningful range of cut-off values in *b_ij_*', the size of the habitat-specific γ-diversity in the Alps was always higher than in the Scandes (Figure 1A). Using the 0.80 threshold of *b_ij_*' as the most conservative, meaningful estimate of the strength of the environmental filter, the size of the habitat-specific γ-diversity was much higher in the Alps (mean: 146; SD: 37) than in the Scandes (mean: 75; SD: 21; one-tailed Student's paired-sample *t*-test, *t* = 33.93, d.f. = 402, *P*≪0.0001; see also Figure 1B) for the environmentally similar Alps-Scandes pairs. Hence, in support of our first hypothesis, we found higher total and habitat-specific γ-diversity in the Alps than in the Scandes.

### Plot-to-plot β-diversity

**Figure 2:**
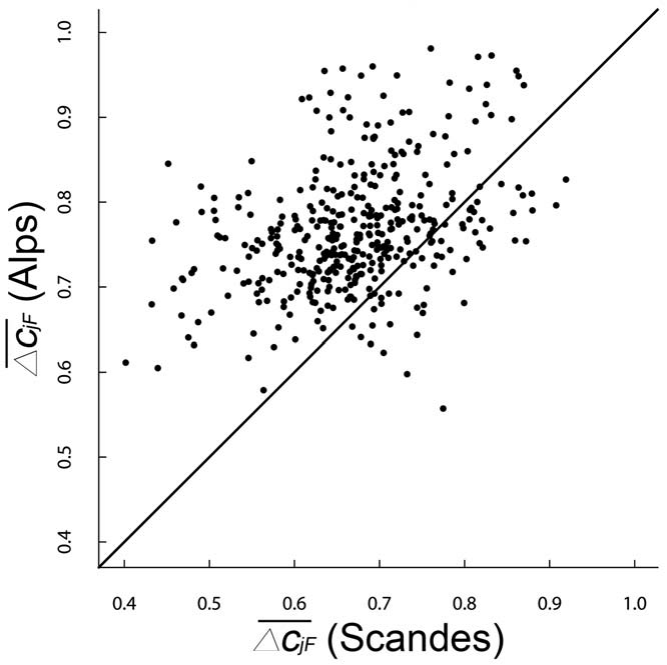
The region effect on plot-to-plot β-diversity. Scatter plots of the average of all of the pair-wise compositional dissimilarity values between each focal sampling plot *F* and all other 402 *j* plots in a given region (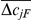), also termed compositional distinctness. Each dot represents one Alps–Scandes pair of environmentally similar plots.

Overall, plot-to-plot β-diversity quantified by pair-wise species turnover (*Δc_jk_*) between all 403 plots within a whole region was clearly higher in the Alps (*Δc_jk_* mean: 0.77, *Δc_jk_* SD: 0.20) than in the Scandes (*Δc_jk_* mean: 0.67; *Δc_jk_* SD: 0.20). Moreover, compositional distinctness, as the average of the *Δc_jF_* values between each focal plot and all other 402 plots in a given region (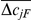), was significantly higher (one-tailed Student's paired-sample *t*-test, *t* = 20.79, d.f. = 402, *P*≪0.0001) in the Alps (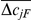 mean: 0.77; 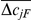 SD: 0.07) than in the Scandes (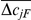 mean: 0.67; 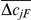 SD: 0.09) (Figure 2) for the environmentally similar Alps-Scandes pairs.

**Figure 3:**
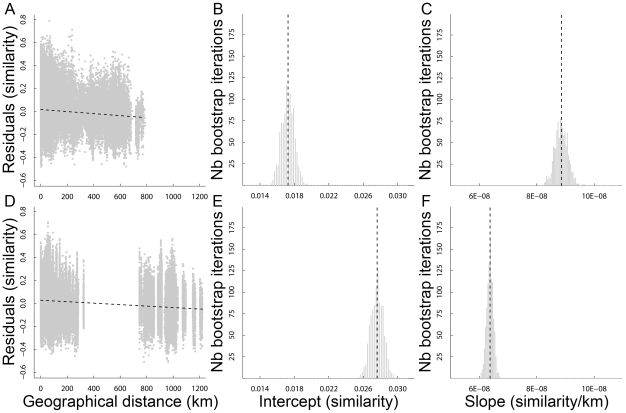
The region effect on the relationship between geographical distance and floristic similarity. Patterns of distance decay of floristic similarity (*sim_jk_*) after accounting for environmental distances between plot pairs in (**A**–**C**) the Alps and (**D**–**F**) the Scandes. (**A**,**D**) Relationship to geographical distance of the residuals of the environmental models in *sim_jk_* and distributions of (**B**,**E**) the intercepts and (**C**,**F**) the slopes yielded by 1000 bootstrap re-samplings of the regression model to test for significant differences between the Alps and the Scandes coefficients. Vertical dotted lines show the original values of the regression coefficients.

The best environmental models for plot-to-plot β-diversity, here represented by floristic similarity (*sim_jk_*), included all six Ellenberg's distance matrices as well as the plot size distance matrix in both regions (Table 1). These seven distance matrices explained 46% and 55% of the plot-to-plot β-diversity variation in the Alps and in the Scandes, respectively. The Pearson correlation coefficient between the residuals from these environmental models and geographical distance was negative in both the Alps (*r* = −0.13, Mantel test *P*<0.0001) and the Scandes (*r* = −0.20, Mantel test *P*<0.0001). However, the regression of the residuals of the environmental models against geographical distance provided quite different coefficients in the two regions (Figure 3). The intercept was lower in the Alps (0.017±SE 0.0007) than in the Scandes (0.028±SE 0.0007) and the difference was significant (*P*<0.001; Figure 3B,E), whereas the slope was higher in the Alps (−8.9×10^−8^±SE 2.2×10^−9^) than in the Scandes (−6.4×10^−8^±SE 1.1×10^−9^) and this difference was also significant (*P*<0.001; Figure 3C,F). Hence, as we expected and even after accounting for differences in the six Ellenberg's distance matrices and the plot size distance matrix, plot-to-plot β-diversity was higher in the Alps than in the Scandes both at small (from 0 to 1 km distance) and increasing geographical distance between plots (up to 1250 km distance).

**Table 1:** Environmental models of floristic similarity in the Alps and the Scandes.

Variable	The Alps (R^2^ adj. = 0.46)			The Scandes (R^2^ adj. = 0.55)		
	Coefficient	Standardized coefficient	*P* value	Coefficient	Standardized coefficient	*P* value
log(dA+1)	−0.01	−0.08	<0.0001	−0.01	−0.07	<0.0001
log(dL+1)	−0.11	−0.22	<0.0001	−0.14	−0.24	<0.0001
log(dT+1)	−0.26	−0.37	<0.0001	−0.20	−0.32	<0.0001
log(dK+1)	−0.11	−0.12	<0.0001	−0.10	−0.12	<0.0001
log(dF+1)	−0.11	−0.18	<0.0001	−0.15	−0.20	<0.0001
log(dR+1)	−0.10	−0.20	<0.0001	−0.08	−0.13	<0.0001
log(dN+1)	−0.06	−0.09	<0.0001	−0.16	−0.23	<0.0001

Regression coefficients from the best environmental model of floristic similarity (*sim_jk_*) against the distance matrices of all six of Ellenberg's indicator factors (dL, dT, dK, dF, dR, and dN) and plot size (dA) for each of the two geographical regions. Significance of the coefficients was estimated by computing 10,000 permutations of the objects of the response distance matrix, namely the floristic similarity matrix, while holding the explanatory distance matrices constant.

### Plot α-diversity

**Figure 4:**
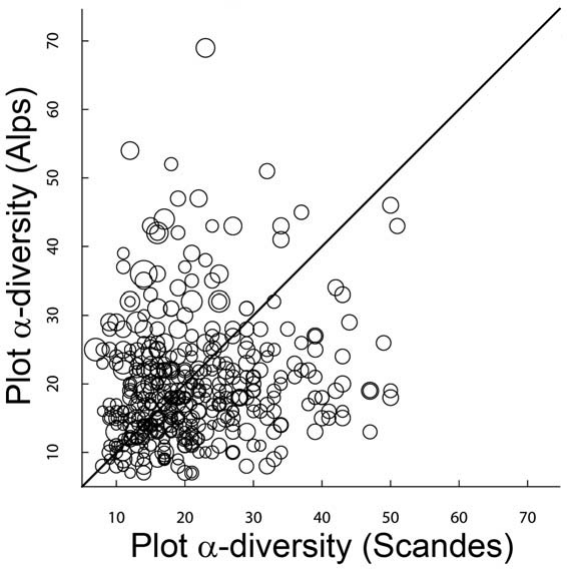
The region effect on plot α-diversity. Each dot represents one Alps–Scandes pair of environmentally similar plots. The size of each dot is proportional to the Ellenberg's R value in the Alps.

Overall, plot α-diversity did not differ between environmentally paired plots in the Alps and the Scandes (Figure 4): plot α-diversity was not significantly higher (one-tailed Student's paired-sample *t*-test, *t* = −1.97, d.f. = 402, *P* = 0.98) in the Alps (mean: 19.92; SD: 8.81) than in the Scandes (mean: 21.07; SD: 9.15). However, we found significant effects of soil pH (R) and plot size (A), as well as a marginal influence of light (L), on Alps–Scandes paired differences in plot α-diversity (Table 2). Among these three variables, R had the greatest effect on plot α-diversity differences, with positive differences (i.e., higher plot α-diversity in the Alps) towards the highest R values (Figure 4), even after accounting for the effect of plot size (Table 2). For pairs with R≥4 in the Alps (moderately acidic to neutral soils; *n* = 97), plot α-diversity was indeed significantly higher (two-tailed Student's paired-sample *t*-test, *t* = 3.71, d.f. = 96, *P* = 0.0003) in the Alps than in the Scandes, whereas for pairs with R<3 in the Alps (strongly acidic soils; *n* = 113), plot α-diversity was significantly lower (two-tailed Student's paired-sample *t*-test, *t* = −5.35, d.f. = 112, *P*<0.0001) in the Alps than in the Scandes. Concerning plot size, for pairs with A≥50 m^2^ in the Alps (larger plots; *n* = 169), plot α-diversity was similar (two-tailed Student's paired-sample *t*-test, *t* = −0.01, d.f. = 168, *P* = 0.99) between the Alps and the Scandes, whereas for pairs with A<5 m^2^ in the Alps (smaller plots; *n* = 168), plot α-diversity was significantly lower (two-tailed Student's paired-sample *t*-test, *t* = −5.04, d.f. = 167, *P*<0.0001) in the Alps than in the Scandes.

**Table 2:** Model of Alps-Scandes paired differences in plot α-diversity.

Variable	Coefficient	Standardized coefficient	Standard error	*t* value
A	0.04	0.23	0.01	4.41
L	2.56	0.20	1.27	2.02
T	0.86	0.04	1.81	0.48
K	−2.02	−0.06	1.78	−1.14
F	−0.27	−0.01	1.06	−0.26
R	3.93	0.31	0.84	4.67
N	−0.13	−0.01	1.28	−0.10

Regression coefficients from the ordinary least square (OLS) model of Alps–Scandes paired differences in plot α-diversity (plot α-diversity in the Alps – plot α-diversity in the Scandes) against the six Ellenberg's indicator factors (L, T, K, F, R, and N) and plot size (A).

Additionally, we found a significant region effect on plot α-diversity in an unpaired ANCOVA of all 806 plots with light (L), temperature (T), soil humidity (F), soil pH (R) and plot size (A) as covariates in the ANCOVA (Table S2). Correcting for spatial autocorrelation in the residuals of this non-spatial model (Figure S3) reduced the region effect and left it only slightly significant, with predicted values from the spatial model in the Scandes (mean: 19.96; SD: 5.27) exceeding those in the Alps (mean: 18.73; SD: 4.30). The residuals in the spatial model were normally distributed and showed no spatial autocorrelation pattern (Figure S3). Thus, contrary to our expectations, plot α-diversity was on average slightly higher in the Scandes than in the Alps even after directly accounting for slight differences in the six Ellenberg's indicator factors and plot size.

### The relationship of plot α-diversity to habitat-specific γ-diversity

**Figure 5:**
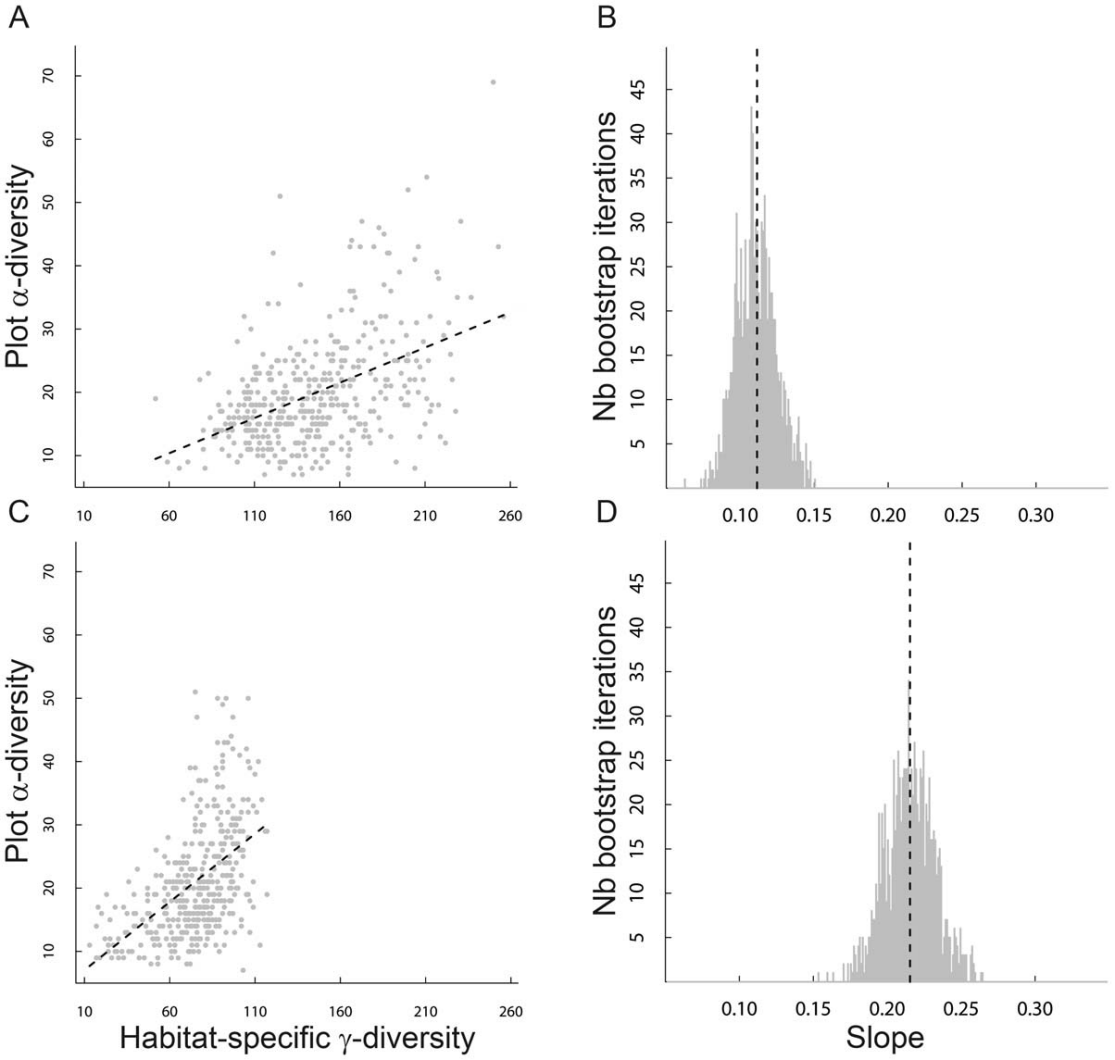
The region effect on the relationship between habitat-specific γ-diversity and plot α-diversity. Relationship of plot α-diversity to habitat-specific γ-diversity in both (**A**,**B**) the Alps and (**C**,**D**) the Scandes: (**A**,**C**) show the relationships based on empirical data and (**B**,**D**) show the distributions of the slope coefficients yielded by 1000 bootstrap randomisations. Note that slopes were plotted with the same range in the *y* axis, thus allowing a direct comparison between regions. Vertical dotted lines show the values of the slope coefficients from empirical data in the Alps and the Scandes. The correlation coefficient *r* between plot α-diversity and habitat-specific γ-diversity was significant (*P*<0.05) in both regions.

We found a significant positive relationship between plot α-diversity and habitat-specific γ-diversity in both the Scandes (*r* = 0.50, *P* = 0.0139) and the Alps (*r* = 0.46, *P* = 0.0461). In contrast, there was no significant positive relationship (*r* = 0.25, *P*>0.9999) between plot α-diversity and habitat-specific γ-diversity for the Alps and the Scandes combined (pooling the data). Additionally, the slope of the regression between plot α-diversity and habitat-specific γ-diversity was lower in the Alps (0.11±SE 0.01; Figure 5A) than in the Scandes (0.21±SE 0.02; Figure 5C), and the difference was significant (*P*<0.001; Figure 5B,D).

## Discussion

### A strong regional effect on total and habitat-specific γ-diversity

Total γ-diversity was approximately two times higher in the Alps than in the Scandes and the comparative analysis of environmentally paired plots in the two regions showed that habitat-specific γ-diversity was also much higher in the Alps than in the Scandes (Figure 1B). These results are consistent with the findings reported by Svenning *et al.* [7] for plant species diversity at a 2500-km^2^ resolution across Europe, a scale that is more representative for γ- than for α-diversity. They further agree with previous findings comparing total γ-diversity between different mountain ranges in Europe and showing that total γ-diversity is much lower in the Scandes than in the Alps and other southern European mountain ranges [61]. More generally, our γ-diversity findings lend further support to the idea that contrasting glacial histories can leave strong legacies in the current species richness at scales larger than 1 km^2^ [10]–[13]. In particular, the region difference on both total and habitat-specific γ-diversity agrees with the hypothesis that higher plant species richness in the Alps than in the Scandes is favoured by two factors. The first is the proximity from numerous peripheral alpine refugia along the border of the Alps [25] and the main glacial refugia in southern Europe [23], [24], offering higher rates of postglacial re-colonisation in the Alps than in the Scandes. The second is topographic heterogeneity [62] within the glaciated central Alps providing ice-free nunatak areas with greater possibilities for *in-situ* survival and diversification [25], [63] compared to the less dissected plateaux within the intensively glaciated Scandes where there is no evidence of continuous *in-situ* survival [27].

In regional comparative studies, where low replication will always pose a limit on our ability to make strong inferences, it is important to consider alternative explanatory mechanisms. The main alternative mechanisms that could generate the regional diversity differences found here involve environmental and area effects. In our analyses we diminished the likelihood that these factors would drive our results by accounting for several of the most important environmental factors (those generally recognized as the most important abiotic niche axes for plants [44]) as well as area effects, thus lending considerable support to a historical interpretation of the observed region differences in total and habitat-specific γ-diversity. Nevertheless, Quaternary history is still not the only possible driver behind the patterns we found. On the one hand, our environmentally similar plot pairs are embedded into landscapes with differing ranges of macroclimatic conditions. As a consequence, the total and habitat-specific γ-diversity of our plots might be larger in the Alps than in the Scandes because the Alps dataset is a subset of an overall regional species pool that extends further to the warm parts of the temperature gradient, thereby representing a wider array of climatic conditions. On the other hand, environmental variables not included as a constraint in our pairing procedure may contribute as well to the regional differences in total and habitat-specific γ-diversity. For instance, patterns of snow distribution are known to be important for alpine plants because individual species vary considerably in their dependence on a protective snow cover or, vice versa, on an early melt out date [64]. It is not possible to completely rule out the effect of such missing variables on the regional differences in total and habitat-specific γ-diversity.

### Plot-to-plot β-diversity also show strong regional differences

Compositional distinctness [30] was higher in the Alps than in the Scandes (Figure 2) as hypothesised from the geologically and geographically more heterogeneous set of refugial re-colonisation sources in the former [25], [65]. Even after correcting for environmental and plot size distances, pair-wise floristic similarity between neighbouring plots (distance<1 km) was significantly lower (Figure 3B,E) and this similarity decreased more strongly with geographical distance in the Alps than in the Scandes (Figure 3C,F). In the case of Europe, the effect of glaciations is an obvious candidate for explaining such differences between regions [56]. Recurrent and rapid climatic shifts throughout Earth's history have caused changes in the geographical distributions of clades, which Dynesius & Jansson [19] designated as orbitally forced species' range dynamics (ORD). The magnitude of ORD varies geographically, being high towards the poles and low towards the equator and thus possibly selecting for vagility [20]. This relationship suggests that traits enhancing vagility (i.e., high dispersal ability and propensity) would be frequently favoured in regions heavily influenced by glacial cycles [20], such as northern Europe where we consistently found a less-distinct species composition of plots and a slower distance decay in floristic similarity than in southern Europe (Figures 2 and 3). Indeed, plot-to-plot β-diversity has previously been found to be negatively related to dispersal ability in North American plant assemblages [66].

Although our findings are consistent with the differential glacial history of the two regions, we again note that there may be alternative explanations despite that we accounted for environmental and area differences among the plots. Notably, differing within-region habitat connectivity constitutes a potentially important alternative driver of differences in the steepness of the distance decay in floristic similarity [67]. Indeed, connectivity between vascular plant communities might be higher in the Scandes than in the Alps: the more complex altitudinal zonation of vegetation, the greater geologic patchiness and the longer history of agricultural land-use in the Alps are more likely to generate island-like distributions of alpine plant communities occurring closer to isolated mountain summits in the Alps. In addition, as discussed in the previous section, missing environmental variables could potentially contribute to the regional differences in plot-to-plot β-diversity and the rate of distance decay in floristic similarity.

The region effect has rarely been explicitly incorporated into analyses that targeted determination of the drivers of β-diversity patterns. To our knowledge, the present study is the first attempt to examine the region effect on pair-wise species turnover anomalies, i.e., differences in plot-to-plot β-diversity patterns in similar environments but with different historical backgrounds. However, the results of some recent work [21], [56], [68] are well in line with our findings. Using country-level inventories, Baselga [56] demonstrated higher spatial turnover in longhorn beetles in southern than in northern Europe. In addition, Graham *et al.* [21] have shown that contemporary patterns of β-diversity across northern Australian rainforests were best explained by Late Quaternary habitat connectivity.

### A small, unexpected regional effect on plot α-diversity

Plot α-diversity was not higher in the Alps than in the Scandes, which contradicts the hypothesised greater plant species richness in the Alps resulting from the region effect. Overall, plot-scale species richness was even somewhat higher in the Scandes, although plot size was slightly larger in the Alps (first quartile: 4 m^2^, median: 30 m^2^, third quartile: 100 m^2^) than in the Scandes (first quartile: 4, median: 25 m^2^, third quartile: 25 m^2^). Our results suggest that regional differences in diversity components at coarse scales (γ-diversity, at ≫1 km^2^, Figure 1B)translate into similar differences at intermediate scales and even at very short distances between plots (β-diversity, at <50 m apart, Figure 3A,D) but tend to disappear or even reverse at the finest scales (α-diversity, at 0.25–450 m^2^, Figure 4). This finding is in line with the general idea that different processes are likely to determine species diversity at different spatial scales [69], [70] and that smaller spatial scales involve processes at shorter temporal scales [70]. Because of the dearth of fine-grained studies, at this point it is unclear whether the scale dependency of the region effect on species diversity identified here is a general phenomenon.

The tendency to find no differences in plot α-diversity between the Alps and the Scandes in our data is unlikely to reflect a methodological artefact involving similarity in species composition between Alps–Scandes pairs. Even if the outcome of the pairing procedure we used involved species compositions as a proxy for environmental conditions, there is a low likelihood that our Alps–Scandes pairs reflect similarity in species composition (only 161 species out of 675 are common to both regions) rather than similarity in environmental conditions. Therefore, Alps–Scandes pairs sharing a similar spectrum of Ellenberg's indicator values do not necessarily share a similar spectrum of species, but might rather show highly distinct species compositions (high pair-wise compositional dissimilarity between environmentally paired plots in the Alps and the Scandes: results not shown). Hence, there is no bias towards selecting for similar plot α-diversity between Alps–Scandes pairs.

In this paper, we also accounted for several of the most important processes affecting plot α-diversity by including both environmental variables and plot size as covariates in our models, thus decreasing the probability that the pattern of plot α-diversity differences between the two regions is due to environmental and area effects. Furthermore, in our analyses of plot α-diversity using a spatial model (Table S2), we corrected the estimated coefficients for spatial autocorrelation that might reflect a missing and spatially autocorrelated environmental variable. Therefore, we consider it unlikely that the pattern of plot α-diversity differences between the two regions is due to environmental processes, although this possibility can never be completely ruled out in regional comparative studies.

Why did the region effect not leave strong differences in plot α-diversity in our environmentally similar Alps–Scandes plot pairs? We can point to at least two alternative and non-exclusive potential mechanisms that might override the predicted glacial history effects: a habitat-connectivity effect and the space-filling limitation. First, we note that most of the plot pairs in our study occurred on acidic soils because vegetation-plot data in the Scandes were largely restricted to the acidic end of the soil pH gradient covered by the Alps data (Figure S1A). This pattern reflects the current relatively rare and patchy distribution of calcareous bedrocks within a matrix of siliceous bedrocks in the Scandes, whereas the areas covered by the two types of bedrock are much more balanced in the Alps [71]. The coarsely heterogeneous and disjunctive distribution of bedrock types in the Alps may act as geological barrier on dispersal, with a major effect on migration pathways during postglacial re-colonisation [65]. In addition, evolved acidic soils were much less common than today and even less common than rejuvenated calcareous soils in the Alps during the Last Glacial Maximum [16], probably causing the extinction of disproportionately more acidophilous than calciphilous species [72], [73]. This historical difference in the extent of acidic and calcareous soils in the Alps, which might reflect the current siliceous-to-calcareous bedrock ratio there, would have favoured calciphilous [74] over acidophilous immigrants after the ice sheet retreat in the Alps. Hence, calciphilous species would have been the first to re-colonise ice-free habitats on young and rejuvenated calcareous soils, providing a strong source of immigrants, whereas geological barriers might have constrained postglacial re-colonisation of acidophilous species in the Alps [65]. As a result, not all of the acidophilous species might have fully re-colonised the areas with current siliceous bedrocks in the Alps. In contrast, postglacial re-colonisation of acidophilous species in the Scandes might have been less constrained by dispersal limitation because of the more continuous distribution of siliceous bedrocks speeding up the appearance of evolved acidic soils and compensating for the lower total γ-diversity in the Scandes. Focusing on the most-acidic soils, we actually fount that plot α-diversity was significantly higher in the Scandes than in the Alps despite the lower total and habitat-specific γ-diversity observed in the Scandes. Thus, plot α-diversity in the Scandes is on average similar or slightly superior to that which we observed in the Alps, reflecting the differing habitat-connectivity structure of geologic features between both regions. Additionally, the longer history of agricultural land-use in the Alps might have fragmented alpine plant communities more than in the Scandes, thus potentially contributing to the unexpected lack of region effect on plot α-diversity.

The second argument we suggest involves plot size as an important feature affecting plot α-diversity. At a given regional extent, substantial differences in plot α-diversity between two environmentally similar areas might be easier to detect at larger than at smaller plot sizes, simply because of the inevitable consequence of the physical limitations of the spatial unit considered and regardless of competition among species [75]. Indeed, space-filling growth of individual plants strongly limits the number of species that can coexist at very small plot sizes. This effect is likely to be even more pronounced in acidic soils where many of the most common species have clonal growth forms, e.g., ericaceous dwarf shrubs such as *Vaccinium myrtillus* (second most frequent species in the Alps data) and *Empetrum nigrum* (third most frequent species in the Scandes data). It should also be noted that few species are physiologically capable of tolerating the high H^+^ concentration and the Al^3+^ toxicity of strongly acidic soils [76]; hence, the limited size of the habitat-specific γ-diversity for such soils is more likely to lead to a saturation of plot α-diversity, regardless of the presence or absence of physical barriers to dispersion. However, as we found significant differences in plot α-diversity for the subset of plots <5 m^2^ towards higher plot α-diversity in the Scandes than in the Alps, any such space restrictions cannot be absolute at the scale of the plots studied.

### The relationship of plot α-diversity to habitat-specific γ-diversity reflects patterns of distance decay in floristic similarity

Plot α-diversity was positively related to habitat-specific γ-diversity in both regions (Figure 5A,C), as proposed by the species pool hypothesis that states that immigration from a regional species pool constrains plot α-diversity [18]. Additionally, the local-to-regional species richness relationship was significantly steeper in the Scandes than in the Alps (Figure 5B,D). As a parallel to a negative relationship between dispersal ability and decrease in site-to-site floristic similarity with respect to site-to-site geographical distance [66], there might be a positive relationship between dispersal ability and increase in plot α-diversity with respect to the size of the habitat-specific γ-diversity. Further work is needed to test this hypothesis, but our results for the plot-to-plot β-diversity component showing steeper distance decay in floristic similarity in the Alps than in the Scandes (Figure 3A,D) are the flipside of our results showing a smaller local-to-regional species richness relationship in the Alps than in the Scandes (Figure 5A,C). We proposed that the steeper distance decay in floristic similarity in the Alps than in the Scandes may reflect selection for high dispersal ability [20] towards northern Europe and greater connectivity between communities [67] in the Scandes than in the Alps. This interpretation may also explain the smaller local-to-regional species richness relationship in the Alps than in the Scandes.

### Conclusion

In this study, we found evidence for strong regional effects on plant community assembly and species diversity patterns in European mountains, although these effects did not necessarily penetrate to affect diversity at the finest (plot) scale. Both total and habitat-specific γ-diversity as well as plot-to-plot β-diversity differed considerably between the Alps and the Scandes even with small distances between plots. The smaller total and habitat-specific γ-diversity, the lower plot-to-plot β-diversity and the reduced distance decay in floristic similarity in the Scandes than in the Alps are all consistent with the idea that divergent regional glacial histories have left strong legacies at scales larger than 1 km^2^. We note that our environmentally similar plot pairs are embedded into landscapes with differing ranges of macroclimatic condition and differing degrees of environmental heterogeneity in the Alps and the Scandes which might contribute to these patterns. By contrast, regional differences in plot α-diversity were weak and inconsistent. Hence, region effects on diversity patterns obviously differ between local and regional/landscape scales. We suggest that the weak regional effect on plot α-diversity may be explained by: (i) the Scandes harbouring relatively more vascular plant species with high dispersal abilities selected after the retreat of the ice sheets and (ii) a higher connectivity between vascular plant communities in the Scandes because of less-complex altitudinal zonation of vegetation, greater continuity in geologic features and younger, less intensive history of agricultural land-use.

## Supporting Information

Text S1 **Details of the procedure to pair environmentally similar communities between the Alps and the Scandes.**
(DOC)

Figure S1 **Distribution of the Alps and the Scandes plots within the environmental space.** Principal component analysis (PCA) of 11,249 plots in the Alps (gray dots) as active and 481 plots in the Scandes (black dots) plotted along the first three principal component (PC) axes. (**A**) PC axes 1 and 2 and (**B**) PC axes 2 and 3 are given, while (**C**) represents a 3D zoom of the first three PC axes showing the 403 Alps–Scandes pairs of plots used in the study. Arrows and their directions indicate increasing values for plot size (A), light (L), temperature (T), continentality (K), soil moisture (F), soil pH (R) and soil fertility (N).
(TIF)

Figure S2 **Comparison of the environmental conditions between the 403 Alps–Scandes pairs.** Boxplots of environmental conditions in both the Alps (Xa, *n* = 403 plots) and the Scandes (Xs, *n* = 403 plots) for the light (L), temperature (T), continentality (K), soil moisture (F), soil pH (R) and soil fertility (N) gradients. The line across the box indicates the median, box boundaries show the interquartile range and whiskers extend up to 1.5 times the interquartile range.
(TIF)

Figure S3 **Spatial autocorrelation in the residuals of the non-spatial and spatial models of the region effect on plot α-diversity.** Correlogram of residuals from the non-spatial model (gray, circles) and the simultaneous autoregressive model with a spatial error model (SAR_err_) (black, squares). Both non-spatial and spatial models have the same relationship between the common logarithm of plot α-diversity and explanatory variables (see Table S2 for details on both models). The spatial weights matrix of SAR_err_ was calculated with a neighbourhood structure involving the 10 nearest neighbours and a row-standardised coding scheme designated as ‘W’ in the R-spdep package [77] in R [53]. Filled symbols display significant Moran's I values (*P*<0.05) whereas open symbols display non-significant values.
(TIF)

Table S1 **Adjusted Ellenberg's indicator values of vascular plants in the Scandes.**
(DOC)

Table S2 **Non-spatial and spatial models of the region effect on plot α-diversity.**
(DOC)
