# Supplementary material for: Cross-Scale Analysis of the Region Effect on Vascular Plant Species Diversity in Southern and Northern European Mountain Ranges
Source: PLoS One. 2010 Dec 22;5(12):e15734. doi: 10.1371/journal.pone.0015734 (PMC3008735; doi:10.1371/journal.pone.0015734)
Supplement: Text S1 — Details of the procedure to pair environmentally similar communities between the Alps and the Scandes. (DOC) [file pone.0015734.s001.doc]

**Text S1. Details of the procedure to pair environmentally similar communities between the Alps and the Scandes.**

The pairing procedure consisted of the following steps:

(i) A centred and scaled principal components analysis (PCA) [1] was conducted on all six of Ellenberg’s indicator factors (L: light, T: temperature, K: continentality, F: soil moisture, R: soil pH and N: soil fertility) [2] and plot size (A) of the 11,249 plots located in the Alps. Plot size was included to control for area effects. The first three principal components accounted for 78% of the total inertia. The first principal component (PC) axis was positively correlated with N and negatively correlated with L (Figure S1A). The second PC axis was negatively correlated with F and positively correlated with T (Figure S1B). The third PC axis was positively correlated to A and K and negatively correlated to R (Figure S1B).

(ii) The 481 plots located in the Scandes were projected onto the first three axes as supplementary plots, i.e., they were passively ordinated (Figure S1).

(iii) To find the plots that were most environmentally similar across the two regions, we focused only on those plots of the Alps that were located within the coordinate range of plots of the Scandes along the first three axes (Figure S1).

(iv) For this subset, we then computed the distances between all cross-region pairs in the 3D PCA space.

(v) Among all the possible Alps–Scandes plot pairs, we then selected the closest one within the 3D PCA space. In an optimisation loop, we subsequently always removed those pairs that already had selected plots included and among the remaining pairs, we selected again the closest pair within the 3D PCA space until a complete set of 481 fully independent Alps–Scandes pairs had been identified.

(vi) Because not all plots had sufficiently close neighbours in the Alps, we removed pairs with distances ≥1 PC unit.

## References

1. Chessel D, Dufour AB, Thioulouse J (2004) The ade4 package - I: One-table methods. R News 4: 5-10.

2. Ellenberg H, Weber HE, Düll R, Wirth V, Werner W, et al. (1992) Zeigerwerte von Pflanzen in Mitteleuropa. Scripta geobotanica 18: 1-248.
