# Supplementary figures and images for: Cross-Scale Analysis of the Region Effect on Vascular Plant Species Diversity in Southern and Northern European Mountain Ranges
Source: PLoS One. 2010 Dec 22;5(12):e15734. doi: 10.1371/journal.pone.0015734 (PMC3008735; doi:10.1371/journal.pone.0015734)

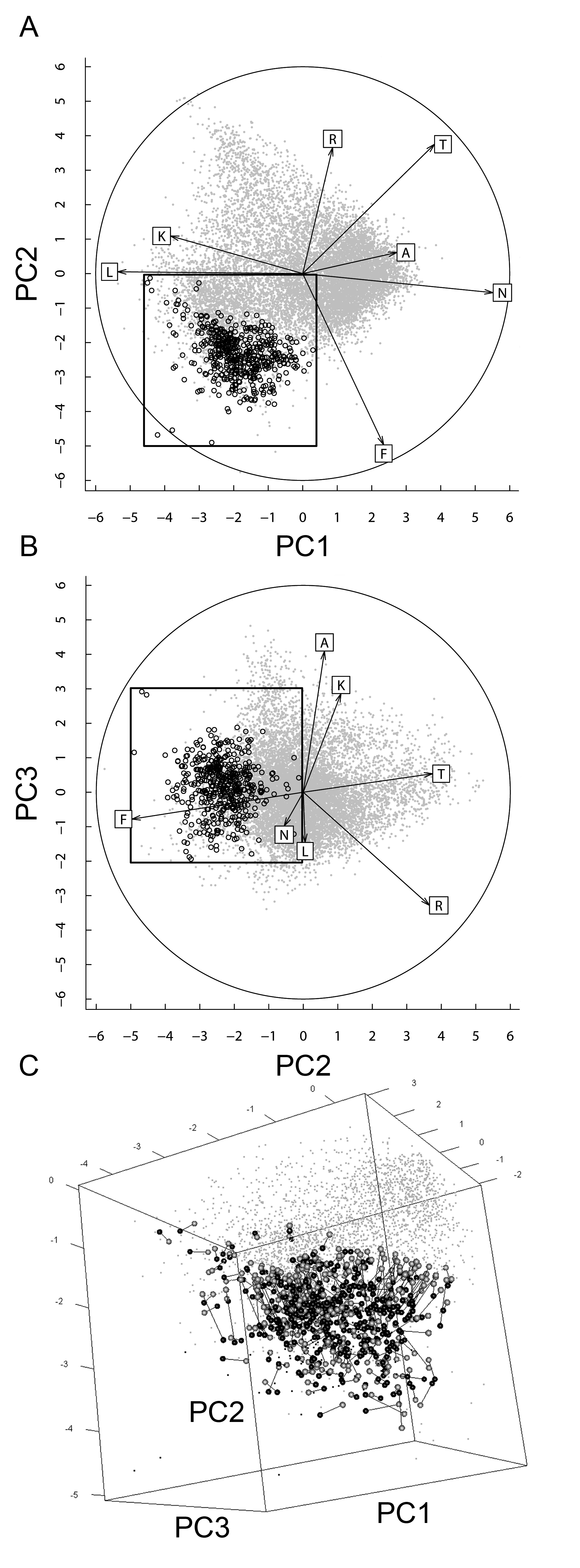

Supplement: Figure S1 — Distribution of the Alps and the Scandes plots within the environmental space. Principal component analysis (PCA) of 11,249 plots in the Alps (gray dots) as active and 481 plots in the Scandes (black dots) plotted along the first three principal component (PC) axes. (A) PC axes 1 and 2 and (B) PC axes 2 and 3 are given, while (C) represents a 3D zoom of the first three PC axes showing the 403 Alps–Scandes pairs of plots used in the study. Arrows and their directions indicate increasing values for plot size (A), light (L), temperature (T), continentality (K), soil moisture (F), soil pH (R) and soil fertility (N). (TIF) [file pone.0015734.s002.tif]

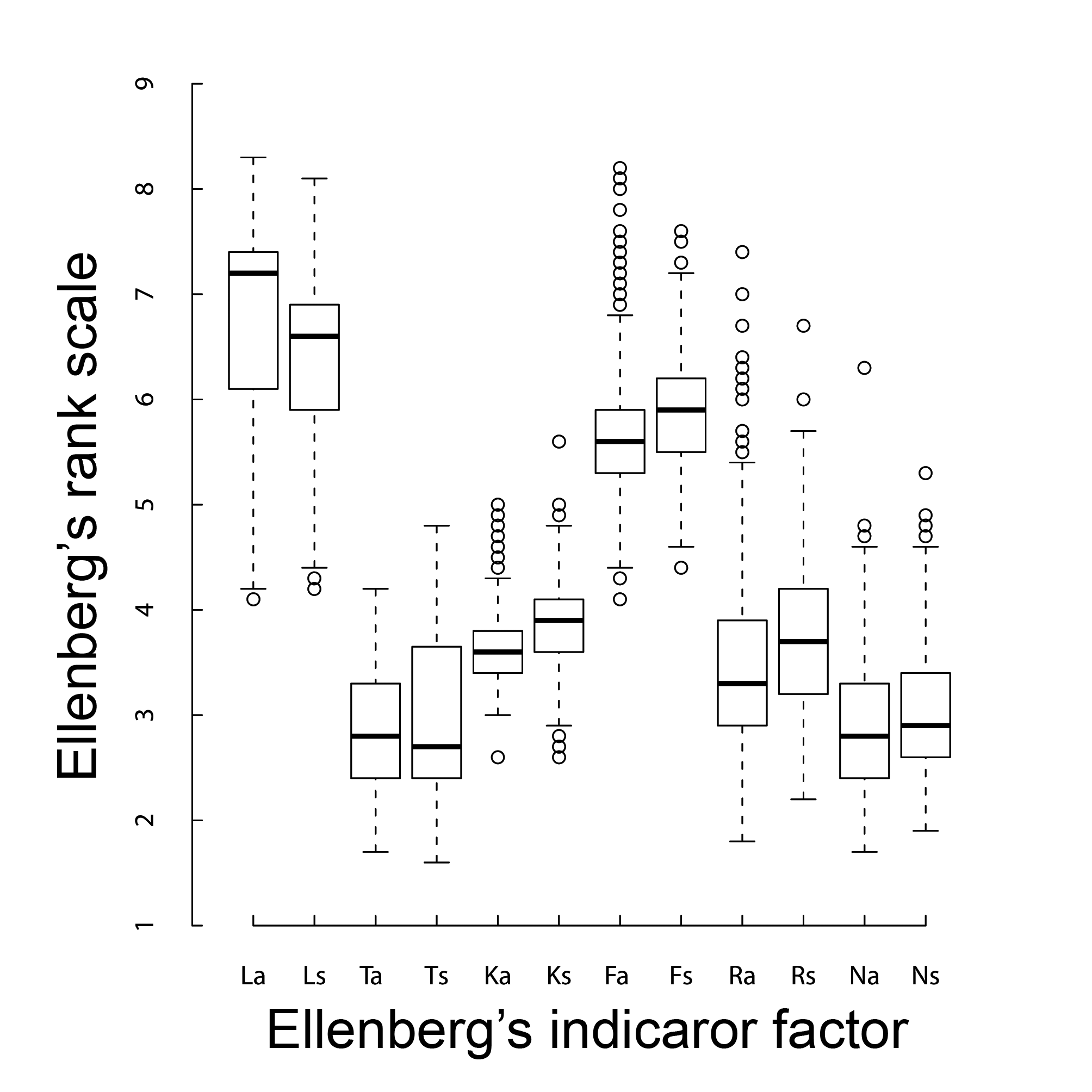

Supplement: Figure S2 — Comparison of the environmental conditions between the 403 Alps–Scandes pairs. Boxplots of environmental conditions in both the Alps (Xa, n = 403 plots) and the Scandes (Xs, n = 403 plots) for the light (L), temperature (T), continentality (K), soil moisture (F), soil pH (R) and soil fertility (N) gradients. The line across the box indicates the median, box boundaries show the interquartile range and whiskers extend up to 1.5 times the interquartile range. (TIF) [file pone.0015734.s003.tif]

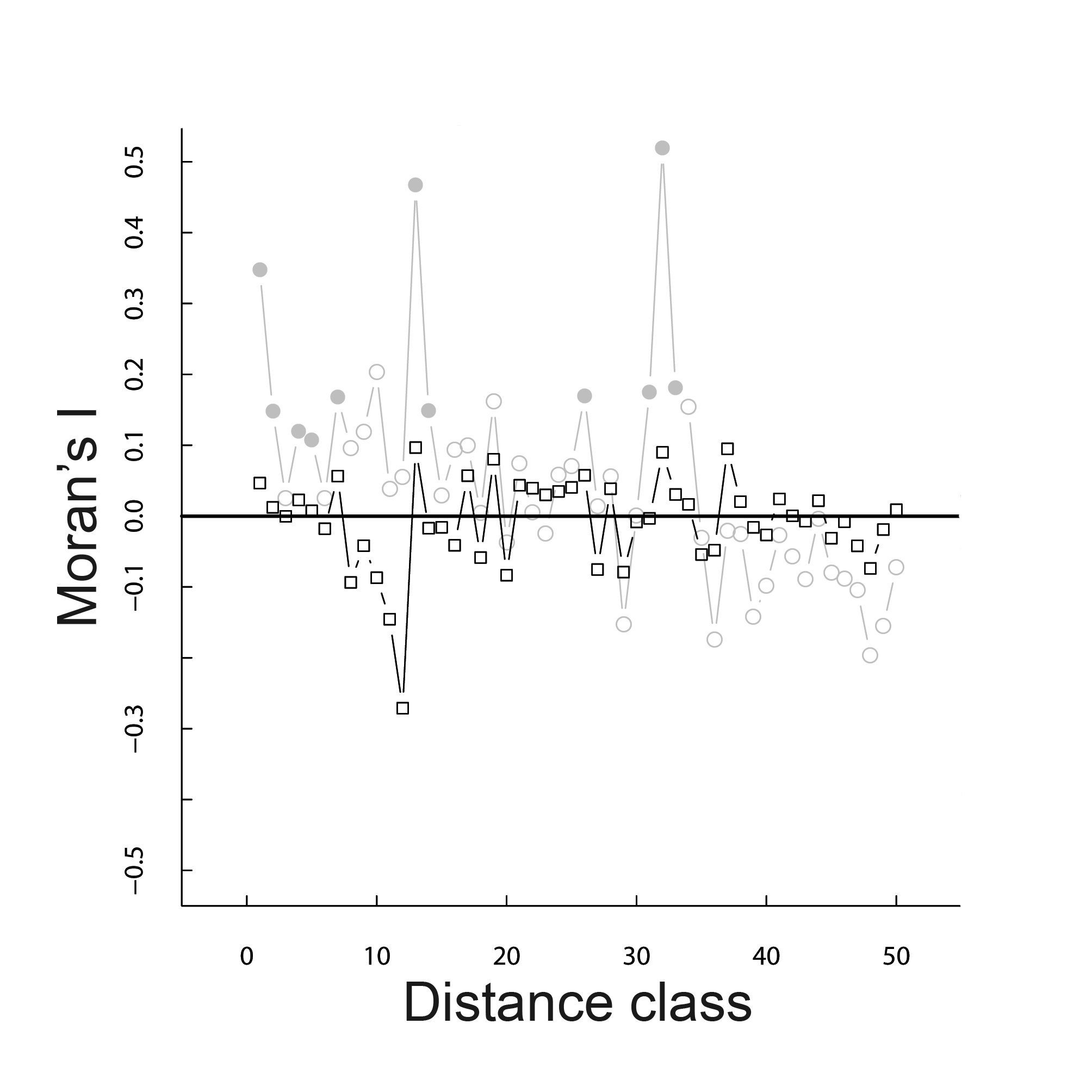

Supplement: Figure S3 — Spatial autocorrelation in the residuals of the non-spatial and spatial models of the region effect on plot α-diversity. Correlogram of residuals from the non-spatial model (gray, circles) and the simultaneous autoregressive model with a spatial error model (SARerr) (black, squares). Both non-spatial and spatial models have the same relationship between the common logarithm of plot α-diversity and explanatory variables (see Table S2 for details on both models). The spatial weights matrix of SARerr was calculated with a neighbourhood structure involving the 10 nearest neighbours and a row-standardised coding scheme designated as ‘W’ in the R-spdep package [77] in R [53]. Filled symbols display significant Moran's I values (P<0.05) whereas open symbols display non-significant values. (TIF) [file pone.0015734.s004.tif]
