# Supplementary material for: Cross-Scale Analysis of the Region Effect on Vascular Plant Species Diversity in Southern and Northern European Mountain Ranges
Source: PLoS One. 2010 Dec 22;5(12):e15734. doi: 10.1371/journal.pone.0015734 (PMC3008735; doi:10.1371/journal.pone.0015734)
Supplement: Table S1 — Adjusted Ellenberg's indicator values of vascular plants in the Scandes. (DOC) [file pone.0015734.s005.doc]

**Table S1. Adjusted Ellenberg’s indicator values of vascular plants in the Scandes.**

| **List of taxa** | **Freq** | **L_or_** | **L_ad_** | **T_or_** | **T_ad_** | **K_or_** | **K_ad_** | **F_or_** | **F_ad_** | **R_or_** | **R_ad_** | **N_or_** | **N_ad_** |
| --- | --- | --- | --- | --- | --- | --- | --- | --- | --- | --- | --- | --- | --- |
| *Agrostis capillaris* | 67 | 7 |  |  |  | 3 | 4 |  | 6 | 4 |  | 4 |  |
| *Alchemilla alpina* | 130 | 9 |  | 2 |  | 2 | 3 | 5 | 6 | 2 | 3 | 2 | 3 |
| *Antennaria dioica* | 83 | 8 | 7 |  | 3 |  | 4 | 4 | 5 | 3 | 5 | 2 | 3 |
| *Anthoxanthum odoratum* | 289 |  |  |  |  |  |  |  | 6 | 5 | 4 |  |  |
| *Arctostaphylos alpinus* | 91 | 7 |  | 2 | 3 | 5 | 4 | 5 | 6 |  |  | 2 |  |
| *Bartsia alpina* | 75 | 8 | 7 | 3 |  | 3 | 4 | 8 | 6 | 7 |  | 3 |  |
| *Betula nana* | 292 | 8 | 7 | 3 |  | 6 |  | 9 |  | 1 |  | 2 |  |
| *Betula pubescens* | 205 | 7 |  |  | 4 |  | 4 | 8 |  | 3 |  | 3 |  |
| *Calluna vulgaris* | 107 | 8 | 6 |  |  | 3 | 4 |  |  | 1 | 2 | 1 | 2 |
| *Campanula rotundifolia* | 112 | 7 |  | 5 |  |  |  |  | 6 |  |  | 2 |  |
| *Carex brunnescens* | 70 | 9 | 8 | 2 | 3 |  |  | 9 |  | 3 |  | 1 | 2 |
| *Carex vaginata* | 136 | 8 | 6 | 3 |  | 6 |  | 9 |  |  |  | 2 |  |
| *Cornus suecica* | 164 | 5 |  | 4 |  | 3 | 4 | 7 |  | 2 | 3 | 2 | 3 |
| *Deschampsia flexuosa* | 624 | 6 |  |  |  | 2 |  |  | 7 | 2 |  | 3 |  |
| *Diphasiastrum alpinum* | 74 | 8 | 7 | 3 |  | 3 |  | 5 |  | 2 | 3 | 2 |  |
| *Dryopteris expansa* | 67 | 4 |  | 3 | 4 |  | 4 | 6 | 7 | 2 | 3 | 2 | 3 |
| *Empetrum nigrum* | 409 | 7 |  |  |  | 3 | 4 | 6 |  |  |  | 2 | 3 |
| *Empetrum nigrum ssp. hermaphroditum* | 181 | 8 |  | 3 |  |  |  | 6 |  | 4 | 2 | 2 |  |
| *Eriophorum vaginatum* | 70 | 7 |  |  |  |  |  | 9 | 8 | 2 |  | 1 | 2 |
| *Euphrasia frigida* | 96 | 7 |  | 3 |  | 3 | 4 | 5 |  | 3 |  | 2 |  |
| *Geranium sylvaticum* | 125 | 6 |  | 4 |  | 4 |  | 6 |  | 6 | 4 | 7 |  |
| *Gymnocarpium dryopteris* | 162 | 3 |  | 4 |  | 5 | 4 | 6 |  | 4 | 3 | 5 |  |
| *Hieracium alpinum* | 72 | 8 | 7 | 2 |  | 3 |  | 5 |  | 1 | 2 | 1 |  |
| *Huperzia selago* | 159 | 4 |  | 3 |  | 3 |  | 6 | 5 | 3 |  | 5 | 3 |
| *Juncus trifidus* | 347 | 8 | 7 | 2 |  | 3 |  | 4 |  | 4 |  | 2 |  |
| *Linnaea borealis* | 114 | 5 |  |  |  | 5 |  | 5 | 6 | 2 | 3 | 2 | 3 |
| *Loiseleuria procumbens* | 171 | 9 | 7 | 2 |  | 3 |  | 5 | 6 | 3 | 2 | 1 | 2 |
| *Luzula multiflora* | 94 | 7 |  |  |  | 4 |  | 5 | 6 | 5 |  | 3 |  |
| *Luzula pilosa* | 70 | 2 | 5 |  | 4 | 3 | 4 | 5 | 6 | 5 | 3 | 4 | 3 |
| *Luzula spicata* | 145 | 8 | 7 | 2 |  | 3 |  | 4 |  | 4 |  | 1 |  |
| *Lycopodium annotinum* | 103 | 3 |  | 4 |  | 3 | 4 | 6 |  | 3 |  | 3 |  |
| *Melampyrum pratense* | 119 |  | 5 |  | 4 | 3 | 4 |  |  | 3 |  | 2 | 3 |
| *Melampyrum sylvaticum* | 70 | 4 |  | 4 |  | 5 | 4 | 5 | 6 | 2 | 4 | 2 |  |
| *Nardus stricta* | 109 | 8 | 6 |  |  | 3 | 4 |  |  | 2 | 3 | 2 | 3 |
| *Omalotheca supina* | 130 | 7 |  | 2 |  | 3 |  | 7 | 6 | 3 | 4 | 4 |  |
| *Oxalis acetosella* | 76 | 1 |  |  | 4 | 3 | 4 | 5 | 6 | 4 |  | 6 |  |
| *Pinus sylvestris* | 70 | 7 | 6 |  |  | 7 | 5 |  | 6 |  |  |  | 2 |
| *Poa alpina* | 79 | 7 | 8 | 3 | 2 | 5 |  | 5 |  |  |  | 7 |  |
| *Polygonum viviparum* | 375 | 7 |  | 2 |  |  |  | 5 |  | 4 |  | 2 | 3 |
| *Potentilla crantzii* | 89 | 9 | 8 | 2 |  | 3 | 4 | 5 |  | 8 |  | 2 | 3 |
| *Pyrola minor* | 103 | 6 |  |  |  |  | 4 | 5 | 6 | 3 | 4 | 2 |  |
| *Rhodiola rosea* | 63 | 7 |  | 4 | 3 | 3 |  | 6 |  | 4 | 5 |  |  |
| *Rumex acetosa* | 208 | 8 |  |  |  |  |  |  |  |  | 4 | 6 |  |
| *Salix herbacea* | 419 | 7 |  | 2 |  | 3 |  | 7 | 5 | 3 |  | 4 |  |
| *Selaginella selaginoides* | 80 | 8 |  | 3 |  | 3 |  | 7 | 6 | 7 | 5 | 3 |  |
| *Sibbaldia procumbens* | 146 | 7 |  | 2 |  | 3 |  | 7 | 6 | 2 | 4 | 4 |  |
| *Silene acaulis* | 167 | 9 | 8 | 1 |  | 3 |  | 4 | 5 | 8 |  | 1 | 3 |
| *Sorbus aucuparia* | 122 | 6 |  |  |  |  | 4 |  |  | 4 | 3 |  |  |
| *Trientalis europaea* | 265 | 5 |  | 5 |  | 7 | 4 |  | 6 | 3 |  | 2 |  |
| *Vaccinium uliginosum* | 353 | 6 |  |  |  | 5 |  |  |  | 1 | 2 | 3 | 2 |
| *Viola biflora* | 117 | 4 |  | 3 |  | 4 |  | 6 | 5 | 7 |  | 6 |  |

List of taxa and their respective frequency (Freq.) in the Scandes (*n* = 1203 plots) along with their original (X_or_) and adjusted (X_ad_) indicator values for the light (L), temperature (T), continentality (K), soil moisture (F), soil pH (R) and soil fertility (N) gradients [1]. Displayed are only taxa that either had no original values (coded as indifferent by Ellenberg *et al.* 1992) and to which new indicator values were assigned or to which adjusted values differed from the original values (see Materials and methods). Empty cells for original values represent indifferent species. Empty cells for adjusted values represent no change and are considered equal to original values. Nomenclature follows Flora Europaea [2].

## References

1. Ellenberg H, Weber HE, Düll R, Wirth V, Werner W, et al. (1992) Zeigerwerte von Pflanzen in Mitteleuropa. Scripta geobotanica 18: 1-248.

2. Tutin TG, Heywood VH, Burges NA, Valentine DH, Walters SM, et al. (2001) Flora Europaea 5 Volume Set and CD-ROM Pack. Cambridge, UK: Cambridge university press. Mixed media. 2392 p.
