# Supplementary material for: Cross-Scale Analysis of the Region Effect on Vascular Plant Species Diversity in Southern and Northern European Mountain Ranges
Source: PLoS One. 2010 Dec 22;5(12):e15734. doi: 10.1371/journal.pone.0015734 (PMC3008735; doi:10.1371/journal.pone.0015734)
Supplement: Table S2 — Non-spatial and spatial models of the region effect on plot α-diversity. (DOC) [file pone.0015734.s006.doc]

**Table S2. Non-spatial and spatial models of the region effect on plot α-diversity.**

| **Variable** | **Non-spatial model (OLS: AIC = 690.7)** | | | | **Spatial model (SAR_err_: AIC = 485.3)** | | | |
| --- | --- | --- | --- | --- | --- | --- | --- | --- |
|  | **Coefficient** | **Standard error** | ***t* value** | ***P* value** | **Coefficient** | **Standard error** | ***z* value** | ***P* value** |
|  |  |  |  |  |  |  |  |  |
| Region | 0.16 | 0.03 | 4.94 | < 0.0001 | 0.14 | 0.07 | 2.16 | 0.0304 |
| A | 0.001 | 0.0003 | 4.85 | < 0.0001 | 0.001 | 0.0003 | 4.33 | < 0.0001 |
| L | 0.16 | 0.03 | 5.84 | < 0.0001 | 0.19 | 0.03 | 7.07 | < 0.0001 |
| T | 0.34 | 0.04 | 9.35 | < 0.0001 | 0.31 | 0.04 | 8.51 | < 0.0001 |
| F | -0.17 | 0.03 | -6.39 | < 0.0001 | -0.16 | 0.02 | -6.46 | < 0.0001 |
| R | 0.17 | 0.02 | 9.74 | < 0.0001 | 0.14 | 0.02 | 8.29 | < 0.0001 |

Analysis of covariance (ANCOVA) of the common logarithm of plot α-diversity in the Alps and the Scandes (*n* = 806 plots) with region as effect. Only significant covariates (A: plot size, L: light, T: temperature, F: soil moisture and R: soil pH) are included in the ANCOVA for both non-spatial and spatial models. OLS refers to the ordinary least square regression of the non-spatial model, and SAR_err_ refers to the simultaneous autoregressive model with a spatial error model. Note that the Aikaike information criterion (AIC) is displayed for both models. The spatial weights matrix of SAR_err_ was calculated with a neighbourhood structure involving the 10 nearest neighbours and a row-standardised coding scheme designated as ‘W’ in the R-spdep package [1] in R [2]. Coefficients are in log units with slope of the region effect displaying log units by which the Scandes exceeds the Alps.

## References

1. Bivand R (2009) spdep: spatial dependence: weighting schemes, statistics and models. R package version 0.4-56.

2. R Development Core Team I (2009) R: A Language and Environment for Statistical Computing. Vienna, Austria: R Foundation for Statistical Computing.
